# Supplementary material for: Vitamin D Receptor (VDR) Gene Polymorphisms Modify the Response to Vitamin D Supplementation: A Systematic Review and Meta-Analysis
Source: Nutrients. 2022 Jan 15;14(2):360. doi: 10.3390/nu14020360 (PMC8780067; doi:10.3390/nu14020360)
Supplement: Supplementary file 1 [file nutrients-14-00360-s001.zip › nutrients-1535803-supplementary.pdf]

Supplementary Material

Table S1. PRISMA checklist

Section/topic	#	Checklist item	Reported on page #
**TITLE**			
Title	1	Identify the report as a systematic review, meta-analysis, or both.	1
**ABSTRACT**			
Structured summary	2	Provide a structured summary including, as applicable: background; objectives; data sources; study eligibility criteria, participants, and interventions; study appraisal and synthesis methods; results; limitations; conclusions and implications of key findings; systematic review registration number.	2
**INTRODUCTION**			
Rationale	3	Describe the rationale for the review in the context of what is already known.	3,4
Objectives	4	Provide an explicit statement of questions being addressed with reference to participants, interventions, comparisons, outcomes, and study design (PICOS).	3,4
**METHODS**			
Protocol and registration	5	Indicate if a review protocol exists, if and where it can be accessed (e.g., Web address), and, if available, provide registration information including registration number.	N/A
Eligibility criteria	6	Specify study characteristics (e.g., PICOS, length of follow-up) and report characteristics (e.g., years considered, language, publication status) used as criteria for eligibility, giving rationale.	4,5
Information sources	7	Describe all information sources (e.g., databases with dates of coverage, contact with study authors to identify additional studies) in the search and date last searched.	4,5
Search	8	Present full electronic search strategy for at least one database, including any limits used, such that it could be repeated.	4,5
Study selection	9	State the process for selecting studies (i.e., screening, eligibility, included in systematic review, and, if applicable, included in the meta-analysis).	4,5
Data collection process	10	Describe method of data extraction from reports (e.g., piloted forms, independently, in duplicate) and any processes for obtaining and confirming data from investigators.	4,5
Data items	11	List and define all variables for which data were sought (e.g., PICOS, funding sources) and any assumptions and simplifications made.	4,5
Risk of bias in individual studies	12	Describe methods used for assessing risk of bias of individual studies (including specification of whether this was done at the study or outcome level), and how this information is to be used in any data synthesis.	4,5
Summary measures	13	State the principal summary measures (e.g., risk ratio, difference in means).	4,5
Synthesis of results	14	Describe the methods of handling data and combining results of studies, if done, including measures of consistency (e.g., I^2) for each meta-analysis.	4,5

Table S1. PRISMA checklist (Continued)

Section/topic	#	Checklist item	Reported on page #
Risk of bias across studies	15	Specify any assessment of risk of bias that may affect the cumulative evidence (e.g., publication bias, selective reporting within studies).	4,5
Additional analyses	16	Describe methods of additional analyses (e.g., sensitivity or subgroup analyses, meta-regression), if done, indicating which were pre-specified.	4,5
**RESULTS**			
Study selection	17	Give numbers of studies screened, assessed for eligibility, and included in the review, with reasons for exclusions at each stage, ideally with a flow diagram.	5-10
Study characteristics	18	For each study, present characteristics for which data were extracted (e.g., study size, PICOS, follow-up period) and provide the citations.	5-10
Risk of bias within studies	19	Present data on risk of bias of each study and, if available, any outcome level assessment (see item 12).	5-10
Results of individual studies	20	For all outcomes considered (benefits or harms), present, for each study: (a) simple summary data for each intervention group (b) effect estimates and confidence intervals, ideally with a forest plot.	5-10
Synthesis of results	21	Present results of each meta-analysis done, including confidence intervals and measures of consistency.	5-10
Risk of bias across studies	22	Present results of any assessment of risk of bias across studies (see Item 15).	5-10
Additional analysis	23	Give results of additional analyses, if done (e.g., sensitivity or subgroup analyses, meta-regression [see Item 16]).	5-10
**DISCUSSION**			
Summary of evidence	24	Summarize the main findings including the strength of evidence for each main outcome; consider their relevance to key groups (e.g., healthcare providers, users, and policy makers).	10-12
Limitations	25	Discuss limitations at study and outcome level (e.g., risk of bias), and at review-level (e.g., incomplete retrieval of identified research, reporting bias).	11,12
Conclusions	26	Provide a general interpretation of the results in the context of other evidence, and implications for future research.	12

Table S2. Reasons for exclusion the articles not included in the meta-analysis

**Authors, year**	**Cause for exclusion**
Ferrari et al, 1998	They did not analyse the effects of vitamin D supplementation. They studied the association of dietary calcium intake and FokI genotypes.
Ferrari et al, 1999	They did not study the effects of vitamin D supplementation. They analysed BMD and calcium and phosphate metabolism according VDR gene SNPs.
Rapauri et al, 2001	It did not evaluate the vitamin D supplementation. They only compared the effects of caffeine intake according VDR gene polymorphisms.
Elenaei et al, 2011	It did not provide vitamin D levels according to genotypes of polymorphisms in the VDR gene. The subjects were classified in "responders", "non-responders" and "controls".
Touvier et al, 2011	It analysed the vitamin D intake, VDR gene SNPs and colorectal cancer risk.
Muindi et al, 2012	It did not provide vitamin D levels according to genotypes of polymorphisms in the VDR gene.
Serrano et al, 2013	It did not provide vitamin D levels according to genotypes of polymorphisms in the VDR gene after follow-up time.
Barry et al, 2014	It only provides the difference in vitamin D levels, not the levels according to VDR gene genotypes before and after the follow-up time.
Correa-Rodriguez et al, 2015	They did not analyse the effects of vitamin D supplementation. They tested the association of VDR gene SNPs and dietary calcium intake.
Chang et al, 2015	They did not analyse the effects of vitamin D supplementation. They determined whether SNPs in VDR and ESR1 genes influence on calcium absorption.
de Medeiros-Cavalcante et al, 2015	They did not provide vitamin D levels according to genotypes of polymorphisms in the VDR gene after follow-up time.
Gaffney-Stomberg et al, 2016	It did not provide vitamin D levels according to genotypes of polymorphisms in the VDR gene after follow-up time.
Normando et al, 2016	They did not provide vitamin D levels according to genotypes of polymorphisms in the VDR gene after follow-up time.
Moradi et al, 2017	It did not provide vitamin D levels according to genotypes of polymorphisms in the VDR gene after follow-up time.
Kazemian et al, 2019	It did not provide vitamin D levels according to genotypes of polymorphisms in the VDR gene after follow-up time.
Hu et al, 2019	It did not provide vitamin D levels according to genotypes of polymorphisms in the VDR gene after follow-up time.
Klahold et al, 2020	They did not provide vitamin D levels according to genotypes of polymorphisms in the VDR gene after follow-up time.
Tomei et al, 2020	They did not provide vitamin D levels according to genotypes of polymorphisms in the VDR gene.
Kazemian et al, 2021	It is a proposal for a protocol for clinical trial.
Brustad et al, 2021	They did not study the response to vitamin D supplementation according VDR gene SNPs. They studied the vitamin D supplementation during pregnancy and VDR SNPs in prevention of offspring persistent wheeze.

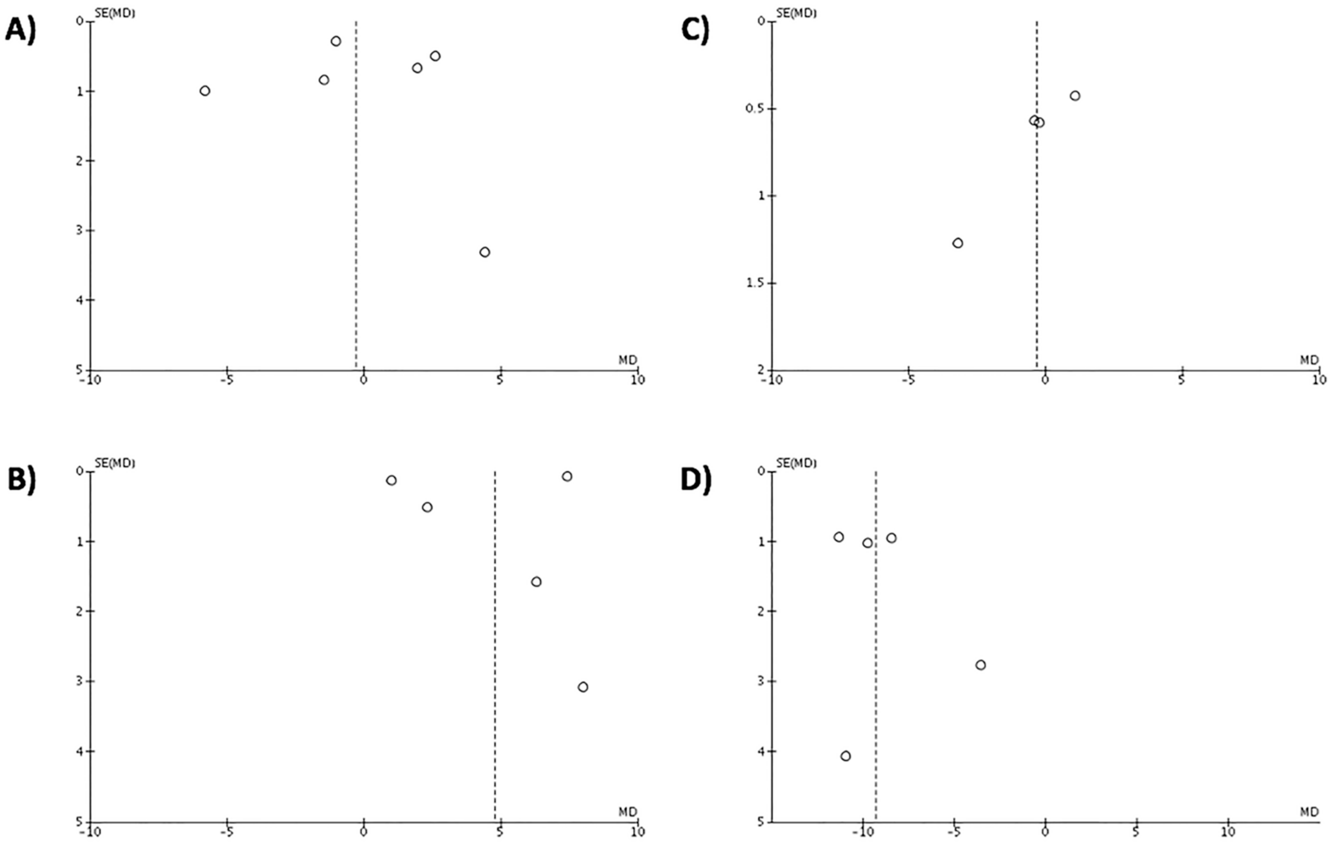

Figure S1. Funnel plot of studies included in the meta-analysis assessing the association of genetic variants in the vitamin D receptor (*VDR*) gene and the response to vitamin D supplementation. (A) BsmI polymorphism. (B) TaqI polymorphism. (C) ApaI polymorphism. (D) FokI polymorphism.
